# Following the money: copy-paste of lifestyle counseling documentation and provider billing

**DOI:** 10.1186/1472-6963-13-377

**Published:** 2013-10-02

**Authors:** Mary Zhang, Maria Shubina, Fritha Morrison, Alexander Turchin

**Affiliations:** 1 Division of Endocrinology, Brigham and Women’s Hospital, 221 Longwood Avenue, Boston, MA 02115, USA; 2 Harvard Medical School, Boston, MA, USA; 3 Quality Performance Management, Partners HealthCare System, Boston, MA, USA

**Keywords:** Electronic medical records, Copy-paste, Cloned documentation, Healthcare costs, Physician billing, Lifestyle counseling

## Abstract

**Background:**

Evidence suggests that copy-pasted components of electronic notes may not reliably reflect the care delivered. Federal agencies have raised concerns that such components may be used to justify inappropriately inflated claims for reimbursement. It is not known whether copied information is used to justify higher evaluation and management (E&M) charges.

**Methods:**

This retrospective cohort study aimed to assess the relationship between the level of evaluation and management (E&M) charges and the method of documentation (none, distinct or copied) of lifestyle counseling (diet, exercise and weight loss) for patients with diabetes mellitus. To determine the association, an ordered multinomial logistic regression model that corrected for clustering within individual providers and patients and adjusted for patient and encounter characteristics was utilized. E&M charge level served as the primary outcome variable. Patients were included if they were followed by primary care physicians affiliated with two academic hospitals for a minimum of two years between 01/01/2000 and 12/13/2009.

**Results:**

Lifestyle counseling was documented in 65.4% of 155,168 primary care encounters of 16,164 patients. Copied counseling was identified in 12,527 encounters. In multivariable analysis higher E&M charges were associated with older patient age, longer notes, treatment with insulin, medication changes and acute complaints. However, copied lifestyle counseling was associated with a decrease of 70.5% in the odds of higher E&M charge levels when time spent on counseling (required to justify higher charges based on counseling) was recorded (p<0.0001). This finding is opposite to what would have been expected if the impetus for copied documentation of lifestyle counseling was an increase in submitted E&M charges.

**Conclusion:**

There is no evidence that copied documentation of lifestyle counseling is used to justify higher evaluation and management charges. Higher charges were generally associated with indicators of complexity of care.

## Background

Electronic medical records (EMRs) can benefit patient care in a number of ways, including enabling timely access to patient information, supporting informed clinical decision-making, improving provider-provider and provider-patient communication and reducing health care costs [1-4]. Utilization of EMRs in the U.S. is increasing and is expected to continue to grow due to strong encouragement by recent federal legislation [5-7].

However, as any tool, EMRs are not always used optimally. In particular concerns have been raised about the inappropriate use of copy and paste to duplicate information across provider notes [8-12]. Studies have estimated that up to 50% of the content in progress notes may be copied from previous documents and found that copying commonly results in documentation errors [13-17]. On the other hand, most providers find the copy-paste feature useful, particularly to increase efficiency of electronic documentation in a time-constrained environment [15]. Errors resulting from copy-paste are therefore thought to primarily be due to negligence.

At the same time, copy-paste could conceivably be used for other purposes as well. For example, in the traditional fee-for-service payment system used by most U.S. physicians, higher charges for a provider-patient encounter can be justified if the provider documents in their note that they spent a certain amount of time with the patient and more than half of that time was spent on counseling the patient. Copy-pasted documentation of counseling could therefore potentially be used to support increased charges to the health insurance (so-called “upcoding”). In fact, in a recent letter to U.S. hospital leadership, the Federal Government expressed strong concern over “troubling indications that some providers are using this technology to game the system” and condemned “cloning of medical records in order to inflate what providers get paid” [11,12]. Under these circumstances, copy-pasted documents not only affect the integrity of the medical record, but may represent health care fraud.

Lifestyle counseling is a critical component of treatment of diabetes [18-22]. It is therefore important to know whether electronic documentation of lifestyle counseling in the records of patients with diabetes is valid. We have previously demonstrated (on a smaller dataset from the same electronic medical record) that copied documentation of lifestyle (diet, exercise and weight loss) counseling, unlike original records, was not associated with improvements in glycemic control in patients with diabetes, and therefore may not always be an accurate representation of the provider-patient encounter [23]. We now conducted a retrospective study of over 16,000 patients with diabetes to determine whether copied lifestyle counseling is being used to justify higher evaluation and management (E&M) charges.

## Methods

### Design

We conducted a retrospective cohort study to investigate whether documentation of lifestyle (diet, exercise and weight loss) counseling that was copied between EMR notes is associated with a higher E&M charge level compared to encounters with no documented counseling.

### Study cohort

Adult patients with diabetes mellitus followed by primary care physicians affiliated with Brigham and Women’s Hospital (BWH) and Massachusetts General Hospital (MGH) for a minimum of two years between 01/01/2000 and 12/31/2009 and had at least one glycated hemoglobin (A1c) ≥ 7.0% were studied. Patients treated by an endocrinologist (identified based on having had more than one encounter with an endocrinologist during the study period) were excluded in order to ensure a single source of diabetes care for the study. The institutional review board at Partners HealthCare System approved the study and the requirement for informed consent was waived.

### Study environment

The study was conducted in practices that utilized Longitudinal Medical Record (LMR) - an ONC-ATCB-certified internally developed EMR. LMR note module has a “Copy” button, which copies the entire note to a new note on the same patient with the current date. LMR also allows users to create patient-independent note templates and custom paragraphs. LMR does not include any decision support for lifestyle counseling. Specifically, LMR does not have any built in check boxes or drop down menu items that could be used to justify higher E&M charge levels based on counseling provided to the patient. All physicians in the practices included in the study are audited annually to ensure compliance with billing regulations. In most practices included in the study, physicians submit the E&M charge level at the time of the patient encounter. Some physicians in the study practices were compensated based on the E&M charges they had submitted and others received a salary. Most physicians who received a salary had an incentive bonus that was based on the E&M charges they submitted. Physician remuneration was not dependent on the their patients’ insurance / insurance payments.

### Study measurements

An individual encounter with a provider in a primary care practice in the setting of an elevated (≥ 7.0%) A1c served as the unit of analysis for the evaluation of the relationship between copied counseling and the E&M charge level. Notes that were dictated (and therefore could not contain any copied documentation) and notes not likely to represent a face-to-face encounter with a physician (e.g. notes with subjects like “medication refill”, “influenza vaccine”) were excluded from consideration (Additional file 1: Table S1).

Documentation of lifestyle counseling was computationally abstracted from the notes, including direct, (e.g. “encouraged daily walking”) and inferred (e.g. “weight has increased since last visit”) instances of lifestyle counseling, as previously described [23]. Lifestyle counseling was inferred if the subject was discussed in a way that made it likely that it was addressed with the patient (e.g. not simply weight recorded in the physical exam section). The natural language processing software used for abstraction of lifestyle counseling was previously validated and had a sensitivity and specificity that ranged between 91–97 and 88-94%, respectively [23].

Lifestyle counseling documented using a sentence that was identical to the sentence used to document the same type of lifestyle counseling in the patient’s previous note by the same provider was considered copied. Notes that had the first record of lifestyle counseling for the patient or lifestyle counseling documentation recorded using a sentence that was different from the previous notes, were considered to have distinct counseling documentation. Notes with no counseling were excluded from determination of the “copied” status.

Notes documenting time spent on direct patient care were identified by computational detection of sentences that included the keywords “spent” and “minutes”. If the sentence also included the word “counseling”, the note was classified as documenting time spent on counseling; if the sentence also included the word “coordination”, the note was classified as documenting time spent on coordination of care.

Most recent A1c and low-density lipoprotein (LDL) measurements within 6 months, and blood pressure (BP) recorded during the encounter were used in the analysis. Most recent body mass index (BMI) measurement for the patient was used in the analysis. Medication intensification was defined as initiation of a new or an increase in the dose of an existing anti-hyperglycemic medication [24]. Anti-hyperglycemic medication intensification was identified from a combination of EMR medication records and computational analysis of narrative provider notes as previously described [25]. The number of medications processed during an encounter was determined as the number of medications that were prescribed, discontinued, or updated during the encounter. Encounters with acute complaints (most commonly acute pain or infection) were identified based on the ICD9 codes associated with the encounter as previously described [24]. Charlson comorbidity index (CCI) was computed using administrative billing codes [26]. Demographic information, weight, height, BP measurements, and medication and laboratory data were obtained from the EMR at Partners HealthCare.

### Study outcome

E&M charge level served as the primary outcome in the analysis. In the United States, all fee-for-service insurance payments for office visits are based on the E&M codes (insurance payments for outpatient visits to all practices in the analysis were based on the fee-for-service model). E&M codes range from level 1 (associated with the lowest payment) to 5 (highest payment). The payments for different E&M code levels vary between different insurance companies and between different localities and providers. The difference between the payments for the highest vs. lowest E&M code levels can be as high as 10-fold.

### Statistical analysis

To determine the association between the presence of copied counseling documentation and the E&M charge level, we constructed an ordered cumulative mixed logistic regression model using GLIMMIX procedure to correct for clustering within individual providers and patients. This model adjusted for the patient’s demographics (age, sex, race, primary language, health insurance, and median income by zip code), most recent BMI, A1c, LDL and BP, acute complaints, treatment with insulin, documentation of anti-hyperglycemic medication intensification, number of medications processed during the encounter and the total number of active medications, length of the note, documentation of time spent on counseling and an interaction term between type of counseling documentation and documentation of time spent on counseling. Average measurements for the patient during the study period were imputed when recent A1c, LDL or BP measurements were not available and an indicator of imputation was also included in the model.

Marginal Cox proportional-hazards model for clustered data [27] was utilized to estimate the association between time to A1c target and the mean monthly number of encounters with copied, distinct or no lifestyle counseling documentation while accounting for clustering within patient-provider pairs. The model was adjusted for the patient’s demographics as well as , inpatient admission during the uncontrolled period, treatment with insulin, PCP encounter frequency, anti-hyperglycemic intensification rate, A1C measurement rate, initial A1C measurement and BMI. When BMI information was not available, mean BMI for the study patients was imputed and an indicator of imputation was included in the model. All analyses were performed with SAS statistical software, version 9.3 (SAS Institute, Inc., Cary, NC).

## Results

We identified 24,097 hyperglycemic adults with diabetes mellitus who were regularly seen by BWH or MGH PCPs. We excluded 7933 patients who were treated by endocrinologists, had no medication records available, only had transient elevations in A1C or suspected A1C measurement errors, or had missing demographic information. The remaining 16,164 patients were included in the study.

The median age of study patients was 59.8 years (Table 1). On average, the patients were followed for 6.2 years during the study period. Study patients did not have their A1C under control >55% of that time, and 53.2% of patients never achieved glycemic control during the study period. On average, patients had 1 to 2 hyperglycemic periods during the study, with a mean initial A1C of 8.3%.

**Table 1:** Patient characteristics

**Variable**	**Value patients with copy-pasted notes**	**Value patients with no copy-pasted notes**
Study patients, n	4,983	11,181
Age*	59.9 (13.2)	60.0 (14.0)
Women, n (%)	2,589 (52.0)	5,680 (50.8)
Ethnicity, n(%)		
White	3,008 (60.4)	6,964 (62.3)
Black	722 (14.5)	1,461 (13.1)
Hispanic	800 (16.1)	1,561 (14.0)
Other (includes unknown)	453 (9.1)	1,195 (10.7)
English is the primary language, n(%)	3,925 (78.8)	9,055 (81.0)
Health insurance, n (%)		
Private	1,801 (36.1)	4,715 (42.2)
Government	3,109 (62.4)	6,302 (56.4)
None	73 (1.5)	164 (1.5)
Charlson comorbidity index	6.8 (4.6)	5.9 (4.6)
Length of follow-up, mean (±SD), mo	82.7 (±28.7)	69.9 (±31.3)
Total time with elevated Hemoglobin A1c, mean (±SD), mo	49.9 (±32.2)	31.1 (±27.5)

Data are mean (SD), unless otherwise indicated.

*Age calculated at the start date of the first uncontrolled period.

Lifestyle counseling was documented on average at 65.4% of primary care encounters during hyperglycemic periods. Most (87.7%) documentations of lifestyle counseling were distinct compared to previous notes by the same provider. Copied counseling records were found much more commonly (76.1:1, p<0.0001) in the notes for the same patient compared to other patients of the same provider, consistent with copy-paste rather than use of templates as the mechanism of their generation.

### Copied counseling documentation and E&M charge level

Over half of patient encounters studied (51.1%) were billed at level 4 E&M charges (Table 2). Encounters with copied documentation of counseling had the highest fraction of level 4 E&M codes at 71.9% (Table 3). Encounters with distinct documentation of counseling had the highest fraction of level 5 codes at 9.6%. In multivariable analysis adjusted for patient and encounter characteristics (Table 4), when time spent on counseling was documented (as required in order to justify the E&M code based on counseling), the cumulative odds of a higher E&M charge level were 70.5% lower for encounters with copied counseling documentation than for encounters with distinct counseling documentation, and 46.1% lower than for encounters with no counseling documentation (p < 0.0001). A sensitivity analysis that did not include note length in the model showed similar results (Additional file 1: Table S2).

**Table 2:** Patient-provider encounter characteristics

**Variable**	**Value**
Total encounters, n	155,168
Encounters with patients on insulin, n (%)	56,253 (36.3)
Most recent hemoglobin A1C, %^†^	8.5 (1.6)
Hemoglobin A1C imputed, n (%)	28,791 (18.6)
Most recent LDL cholesterol, mg/dL^†^	96.5 (32.1)
LDL imputed, n (%)	72,914 (47.0)
SBP, mm Hg	130.3 (17.4)
DBP, mmHg	74.3 (10.8)
BP imputed, n (%)	14,840 (9.6)
Most recent BMI, kg/m^2*^	32.9 (6.6)
BMI imputed, n (%)	15,072 (9.7)
Acute complaints, n (%)	52,203 (33.6)
Total active medications	8.2 (5.0)
Medications processed during the encounter	1.8 (2.5)
Anti-hyperglycemic medication intensification, n (%)	20,586 (13.3)
Note length, 1000s of characters	2.9 (2.2)
Lifestyle counseling, n (%)	
Distinct	88,970 (57.3)
Copied	12,527 (8.1)
None	53,671 (34.6)
Visit category, n (%)	
Physical	4,863 (3.1)
Follow-up	150, 305 (96.9)
Documentation of time spent, n (%)	
Counseling	3,406 (2.2)
Coordination of care	245 (0.2)
Other	3361 (2.2)
None	148,156 (95.5)
Billing level, n (%)	
1	4062 (2.6)
2	5151 (3.3)
3	54,304 (35.0)
4	79,258 (51.1)
5	12,393 (8.0)

Data are mean (SD), unless otherwise indicated.

*Recorded closest in time to encounter.

†Most recent measurement taken within 6 months prior to the encounter.

**Table 3:** E&M charge level by counseling type

**Level**	**Distinct counseling**	**Copied counseling**	**No counseling**
1	2,132 (2.4)	77 (0.6)	1,853 (3.5)
2	2,464 (2.8)	70 (0.6)	2,617 (4.9)
3	28,766 (32.3)	2,480 (19.8)	23,058 (43.0)
4	47,033 (52.9)	9,004 (71.9)	23,221 (43.3)
5	8,575 (9.6)	896 (7.2)	2,922 (5.4)
Total	88,970 (100)	12,527 (100%)	53,671 (100)

Data are frequency (%).

**Table 4:** Effects of encounter and patient characteristics on E&M charge level

**Variable**	**Estimate**	**95% Confidence limits**		**P- value**	**Odds ratio**
Physical^1^	−2.59	−2.66	−2.52	<.0001	0.0753
Income ($1,000)	−0.00245	−0.00413	−0.000770	0.00440	0.998
Female^2^	−0.127	−0.183	−0.0714	<.0001	0.881
Caucasian^3^	0.653	0.589	0.718	<.0001	1.92
Government Insurance^4^	−0.335	−0.397	−0.273	<.0001	9.715
English is the primary language^5^	−0.189	−0.258	−0.120	<.0001	0.828
Age (Decade)	0.214	0.189	0.239	<.0001	1.24
Hemoglobin A1C (over 7%)^†^	−0.0170	−0.0277	−0.00619	0.00200	0.983
Hemoglobin A1C imputed^6^	0.275	0.240	0.310	<.0001	1.32
LDL cholesterol (over 100 mg/dL)^†^	0.0000170	−0.000820	0.000851	0.967	1.00
LDL imputed^7^	0.0513	0.0237	0.0788	0.000300	1.05
SBP (over 130 mmHg)	0.000884	−0.000430	0.00220	0.187	1.00
DBP (over 85 mmHg)	0.00230	−0.00210	0.00669	0.306	1.00
BP imputed^8^	−1.06	−1.10	−1.01	<.0001	0.347
CCI	−0.00524	−0.0124	0.00196	0.154	0.995
Total Active Medications	−0.00282	−0.00670	0.00105	0.153	0.997
BMI (over 25)^*^	0.00352	−0.000850	0.00789	0.114	1.00
BMI Imputed^9^	−0.0369	−0.127	0.0529	0.420	0.964
Anti-hyperglycemic medication intensification^§^	0.210	0.174	0.247	<.0001	1.23
Medications updated during the encounter	0.108	0.102	0.113	<.0001	1.11
Treatment with Insulin^10^	0.0562	0.0126	0.0998	0.0115	1.06
Note length (log)	0.793	0.773	0.814	<.0001	2.21
Acute complaints^11^	0.277	0.249	0.304	<.0001	1.32
Distinct counseling documentation^‡12^	0.272	0.242	0.302	<.0001	1.31
Copied counseling documentation^‡12^	0.336	0.276	0.395	<.0001	1.40
Documentation of time spent counseling^13^	1.37	1.11	1.63	<.0001	3.94
Interaction between documentation of time spent counseling & distinct counseling^‡^	0.330	0.0527	0.607	0.0196	1.39
Interaction between documentation of time spent counseling & copied counseling^‡^	−0.954	−1.31	−0.592	<.0001	0.39

*Recorded closest in time to encounter.

†Most recent measurement taken within 6 months prior to the encounter.

‡Encounters with no counseling documentation served as the reference.

^1^Reference category: encounter was billed as a follow-up visit.

^2^Reference category: male.

^3^Reference category: non-Caucasian.

^4^Reference category: private health insurance.

^5^Reference category: primary language other than English.

^6^Reference category: data available for hemoglobin A1c.

^7^Reference category: data available for LDL (low-density lipoprotein) cholesterol.

^8^Reference category: data available for blood pressure (BP).

^9^Reference category: data available for body mass index (BMI).

^10^Reference category: patients not treated with insulin.

^11^Reference category: no acute complaints documented for the encounter.

^12^Reference category: no lifestyle counseling documented.

^13^Reference category: time spent counseling not documented.

A1C= Glycated hemoglobin. LDL=Low-density lipoprotein. SBP= Systolic blood pressure. DBP= Diastolic blood pressure. BP= Blood pressure. CCI= Charlson comorbidity index. BMI=Body mass index.

## Discussion

As EMRs are being deployed more widely, concerns have been raised that they can also be used to obtain revenue gains through a variety of mechanisms including increased billing coding levels. In particular, a recent report cited several hospitals that increased the share of highest-paying insurance claims by 40 to 80% soon after they introduced electronic health records [11]. A particular concern has been expressed over the use of “cloned” documentation that is copy-pasted from the older records of the same patient or from records of other patients, but may not accurately reflect the care actually delivered.

Copy-pasted documentation could be used to justify higher billing charges in several ways. It may be used to describe review of systems and / or physical examination to fulfill the requirements for higher E&M codes based on the number of organ systems examined. Alternatively, it may be used to “clone” descriptions of counseling provided to the patient to enable E&M charges based on the time spent with the patient. Therefore, in the light of a recent finding that copied documentation of lifestyle counseling may not always accurately represent the care delivered [23], we sought to determine whether it could have been used to generate higher revenue.

In this large retrospective study we did not find evidence that copied documentation of lifestyle counseling was utilized to raise the level of E&M charges. Reimbursement for lifestyle counseling requires documentation of time spent on counseling. When documentation of time spent on counseling was present in EMR notes containing documentation of lifestyle counseling, distinct counseling was associated with a significant increase in the E&M charge level, as expected. On the other hand, when time spent on counseling was recorded in notes with copied lifestyle counseling, a marked decrease in E&M charge level was observed. The most likely explanation for this finding is that both documentation of lifestyle counseling and time spent on counseling were copied from a previous note, but the E&M charge level reflected the care actually delivered.

In the absence of documentation of time spent counseling, copied counseling documentation was associated with slightly higher charges. This finding may reflect the influence that other patient and treatment characteristics can have on documentation behavior and E&M charges. Copying could be more common for complex patients whose historical information has to be repeatedly documented in every note. At the same time, complex patients would also be more likely to incur higher E&M charges. Our other findings are consistent with this explanation: higher E&M charges were associated with many measures of increased patient complexity including greater patient age, primary language other than English, treatment with insulin, presence of acute complaints, anti-hyperglycemic medication intensification, number of medications processed, and longer note length. These findings offer strong evidence that, on average, E&M charge levels reflect the complexity of the care delivered.

Nevertheless, even if the copy-paste feature is not systematically used to justify higher E&M charges, this does not exclude the possibility that individual providers may use it for that purpose. A recent report found, for example, that not only have Medicare payments for E&M services increased by 48% between 2001 and 2010, but the consistent billing of higher level E&M codes by physicians representing less than 1% of 442,000 physicians nationwide cost Medicare as much as $108 million annually [28]. Therefore measures to ensure compliance with E&M coding regulations could include interventions aiming to minimize the inappropriate copy-paste of electronic documentation. These could include educational interventions for physicians. In a survey of physicians at medical centers using computerized documentation systems, over 90% of participants expressed the need for training and education on the responsible use of the copy-paste feature [15]. Another approach that has anecdotally been successful at several institutions is to present copied fragments of the notes in a different color from the rest of the text. By making the “cloned” text immediately and permanently apparent, this approach may both decrease inappropriate utilization of copy-paste and facilitate monitoring and auditing.

Our study had several limitations. Primary data were obtained from an internally developed EMR system. However, copy paste is a common feature of EMR systems and not likely to be unique to the systems utilized in the study, as evidenced by numerous studies describing the ramifications of copied electronic documentation [8,9,16,17]. Direct evidence that lifestyle counseling documented in the note was copied was not available for the analysis. However, strong indirect evidence was provided, including demonstration of much higher prevalence of copied sentences within the same patient compared to other patients of the same provider. This finding is consistent with the functionality of the Copy button in the EMR used in this study which copies the entire note within the same patient. Templates, which can be used across different patients, would have led to a more uniform distribution of copied records. To establish that lifestyle counseling documentation was copied, we required that it be exactly identical to documentation found in the previous note. Though this approach may underestimate the true prevalence of copying as the copied text is frequently subsequently altered, altering copied text requires significantly greater cognitive involvement on the part of the author and may be more likely to reflect the patient-provider encounter. Our analytical approach would not be able to detect a small number of healthcare providers that were using the copy-paste feature for documentation of lifestyle counseling to justify increased E&M charge levels. Our study was conducted in practices affiliated with two academic medical centers, thus limiting generalizability of results to private practices. This limitation may have been ameliorated by the productivity incentives implemented in many of the primary care practices in these medical centers, potentially leading to documentation behaviors similar to private practices. At the same time, some patients may have seen physicians outside of the study practices during the study period. Retrospective nature of the study does not establish causality in the associations that were found in the study. However, it is unlikely that a randomized study on copied clinical documentation would be conducted in the future.

## Conclusion

In summary, we have not found evidence that that copied documentation of lifestyle counseling is used to justify higher E&M charges. Instead, higher charges were associated with a number of markers of patient and encounter complexity, indicating that most providers are compliant with the spirit of insurance regulations.

## Competing interests

The authors declare that they have no competing interests.

## Authors’ contributions

AT and FM obtained data. MZ and FM conducted data analysis. MS supervised statistical analysis. MZ drafted the manuscript. AT obtained funding and supervised the project. All authors critically reviewed the manuscript and approved the final version.

## Pre-publication history

The pre-publication history for this paper can be accessed here:

http://www.biomedcentral.com/1472-6963/13/377/prepub

## Supplementary Material

Additional file 1: Table S1 Notes excluded from the analysis. Table S2. Effects of encounter and patient characteristics on E&M charge level without length of encounter note as an explanatory variable. A1C= Glycated hemoglobin. LDL=Low-density lipoprotein. SBP= Systolic blood pressure. DBP= Diastolic blood pressure. BP= Blood pressure. CCI= Charlson comorbidity index. BMI=Body mass index. Click here for file
