# Supplementary material for: Following the money: copy-paste of lifestyle counseling documentation and provider billing
Source: BMC Health Serv Res. 2013 Oct 2;13:377. doi: 10.1186/1472-6963-13-377 (PMC3850686; doi:10.1186/1472-6963-13-377)
Supplement: Additional file 1: Table S1 — Notes excluded from the analysis. Table S2. Effects of encounter and patient characteristics on E&M charge level without length of encounter note as an explanatory variable. A1C= Glycated hemoglobin. LDL=Low-density lipoprotein. SBP= Systolic blood pressure. DBP= Diastolic blood pressure. BP= Blood pressure. CCI= Charlson comorbidity index. BMI=Body mass index. [file 1472-6963-13-377-S1.docx]

**Additional file**

**Table S1. Notes excluded from the analysis.**

| Reason for exclusion | Number of notes |
| --- | --- |
| Dictated | 58,046 |
| Subject: “phone” | 2,415 |
| Subject: “letter” | 1,391 |
| Subject: “medication refill” | 349 |
| Subject: “Influenza” | 314 |
| Subject: “POCT results/FSBS” | 284 |
| Subject: “Flu shot” | 263 |
| Subject: “Vit B12 injection” | 240 |
| Subject: “MGH care management note” | 145 |
| Subject: “Call” | 141 |
| Subject: “Pneumovax” | 102 |
| Subject: “No show” | 93 |
| Subject: “Influenza vaccine” | 86 |
| Subject: “Social work” | 69 |
| Subject: “Referral form” | 27 |
| Subject: “Family social history” | 13 |
| Subject: “Result manager” | 7 |
| Subject: “Rheumatology follow up visit” | 2 |
| Subject “Gyn” | 1 |

**Table S2. Effects of encounter and patient characteristics on E&M charge level without length of encounter note as an explanatory variable.** A1C= Glycated hemoglobin. LDL=Low-density lipoprotein. SBP= Systolic blood pressure. DBP= Diastolic blood pressure. BP= Blood pressure. CCI= Charlson comorbidity index. BMI=Body mass index.

| **Variable** | **Estimate** | **95% Confidence Limits** | | **P- value** | **Odds Ratio** |
| --- | --- | --- | --- | --- | --- |
| Physical^§^ | -2.20 | -2.27 | -2.13 | <.0001 | 0.111 |
| Income ($1000) | -0.00218 | -0.00381 | -0.000540 | 0.00930 | 0.998 |
| Female^§^ | -0.115 | -0.169 | -0.0612 | <.0001 | 0.891 |
| Caucasian^§^ | 0.651 | 0.588 | 0.715 | <.0001 | 1.92 |
| Government Insurance^§^ | -0.380 | -0.441 | -0.320 | <.0001 | 0.684 |
| English is the primary language^§^ | -0.120 | -0.187 | -0.0524 | 0.0005 | 0.887 |
| Age (Decade) | 0.269 | 0.244 | 0.293 | <.0001 | 1.31 |
| Hemoglobin A1C (over 7%)^†^ | -0.0241 | -0.0347 | -0.0135 | <.0001 | 0.976 |
| Hemoglobin A1C imputed^§^ | 0.304 | 0.269 | 0.338 | <.0001 | 1.35 |
| LDL cholesterol (over 100 mg/dL)^†^ | -0.000280 | -0.00110 | 0.000537 | 0.498 | 1.00 |
| LDL imputed^§^ | 0.0401 | 0.0130 | 0.0673 | 0.0038 | 1.04 |
| SBP (over 130 mmHg) | 0.000419 | -0.000880 | 0.00171 | 0.526 | 1.00 |
| DBP (over 85 mmHg) | 0.00289 | -0.00145 | 0.00723 | 0.192 | 1.00 |
| BP imputed^§^ | -1.32 | -1.36 | -1.27 | <.0001 | 0.268 |
| CCI | -0.0131 | -0.0201 | -0.00604 | 0.0003 | 0.987 |
| Total Active Medications | 0.00836 | 0.00455 | 0.0122 | <.0001 | 1.01 |
| BMI (over 25)^*^ | 0.00491 | 0.000655 | 0.00917 | 0.0237 | 1.00 |
| BMI Imputed^§^ | -0.0515 | -0.139 | 0.0358 | 0.248 | 0.950 |
| Anti-hyperglycemic medication intensification^§^ | 0.232 | 0.196 | 0.268 | <.0001 | 1.26 |
| Medications updated during the encounter | 0.115 | 0.109 | 0.120 | <.0001 | 1.12 |
| Treatment with Insulin^§^ | 0.120 | 0.0775 | 0.163 | <.0001 | 1.13 |
| Acute complaints^§^ | 0.311 | 0.284 | 0.338 | <.0001 | 1.37 |
| Distinct counseling documentation^‡§^ | 0.616 | 0.588 | 0.645 | <.0001 | 1.85 |
| Copied counseling documentation^‡§^ | 0.804 | 0.747 | 0.861 | <.0001 | 2.23 |
| Documentation of time spent counseling^§^ | 1.66 | 1.41 | 1.92 | <.0001 | 5.28 |
| Interaction between documentation of  time spent counseling & distinct counseling^‡^ | 0.254 | -0.0194 | 0.528 | 0.0686 | 1.29 |
| Interaction between documentation of  time spent counseling & copied counseling^‡^ | -1.11 | -1.46 | -0.750 | <.0001 | 0.331 |

*Recorded closest in time to encounter

†Most recent measurement taken within 6 months prior to the encounter

‡Encounters with no counseling documentation served as the reference

§Categorical variable
